# Idiopathic Granulomatous Mastitis Presenting as a Breast Pseudotumor: Case Reports with Review of the Literature

**DOI:** 10.1155/2018/4264012

**Published:** 2018-12-27

**Authors:** Nour Abdul Halim, Imad Uthman, Rayan Rammal, Hazem I. Assi

**Affiliations:** ^1^Division of Hematology and Oncology, Department of Internal Medicine, American University of Beirut, Beirut, Lebanon; ^2^Division of Rheumatology, Allergy, and Immunology, Department of Internal Medicine, American University of Beirut, Beirut, Lebanon; ^3^Division of Anatomic Pathology, Department of Pathology, American University of Beirut, Beirut, Lebanon

## Abstract

Idiopathic granulomatous mastitis is a rare benign inflammatory breast disease that affects women of childbearing age with a history of breastfeeding. It usually presents as an enlarging breast mass that can greatly mimic breast cancer. Moreover, it does not have a specific radiographic finding, so the only way to reach a definitive diagnosis is by core biopsy and histology. Furthermore, a consensus regarding the best treatment modality has not been reached yet. In this report, we describe the cases of two patients who suffered from this disease, and to our knowledge, such a report is the first of its kind to address this topic in this region. Therefore, because of its uncommon nature and obscure presentation, we hereby report two cases of idiopathic granulomatous mastitis. The clinical presentation, treatment, and pathological findings are described, and a literature review on idiopathic granulomatous mastitis will be reported.

## 1. Introduction

This case report is the first of its kind in the Middle East region, as no previous articles have reported such cases of idiopathic granulomatous mastitis in this area before. The obscure presentation and great degree of radiographic overlap with breast cancer further increase the uniqueness and importance of this report.

Idiopathic granulomatous mastitis (IGM) was first reported in 1972 [1], by Kessler and Wolloch [2]. It is described as an uncommon, benign inflammatory breast disease of unknown etiology [1, 3]. It most commonly presents in parous women of reproductive age [1, 4, 5], with a history of breastfeeding [2] in the previous 5-6 years [6]. It mainly presents as an enlarging breast mass, which can be associated with pain and lymphadenopathy [7]. Nipple retraction and skin retraction, as well as nipple discharge, have also been reported, but are less common findings. This mass or lump can be later complicated by abscess formation [5]. GM may be treated with expectant management, a systemic therapy which includes a combination of oral and topical steroids, immunosuppressants, or surgery [7]. The main aim of therapy is palliation of symptoms. Granulomatous mastitis will usually take 1-2 years to resolve on its own if left untreated [7]. The disease most frequently appears in the 3rd or 4th decade of life [6], and reports have shown that the youngest female diagnosed with GM was 11 years and the oldest was 83 years [8].

## 2. Case Presentations

The first case we present is that of a 29-year-old woman who presented to the clinic with the complaint of an enlarging left breast mass. She first noticed that this mass almost 2 years ago and mentions that it has been growing in size and becoming more erythematous and tender. She did not report any recent weight loss or change in appetite. She is married with 2 children, and she does not have any illnesses. She smokes hubble-bubble almost 4 times per week and does not drink alcohol. Surgical history is significant for 2 previous Cesarean sections with no complications. History of her current illness dates back to June 2016 when the patient felt a mass in her left breast; upon further investigation, she was diagnosed with idiopathic granulomatous mastitis and later (December 2017) developed an abscess that drained on its own. She was initially treated with methotrexate and later switched to prednisone and mycophenolate with minimal improvement. At the clinic, her vitals were within normal limits, and on physical examination, there was a left breast lump found at the upper inner quadrant with some erythema and inflammation surrounding it. Moreover, there was some skin retraction in this area.

Core biopsy done at an outside hospital in June 2017 revealed no granulomas. Ultrasound done at that time showed a persistent ill-defined hypoechoic mass that appeared initially subdermally and was spanning more than 4 × 1.4 cm. Moreover, multiple deeper masses were seen, one of which was not located within the breast measuring 12.3 × 8.5 mm. Axillary nodes were insignificant and not well appreciated on imaging.

Fine-needle aspirate done in July 2017 was negative for malignancy and was reported to have abscess formation. The slides revealed a heavy inflammatory infiltrate predominantly composed of polymorphonuclear leukocytes. No ductal epithelial cells were seen.

**Figure 1:**
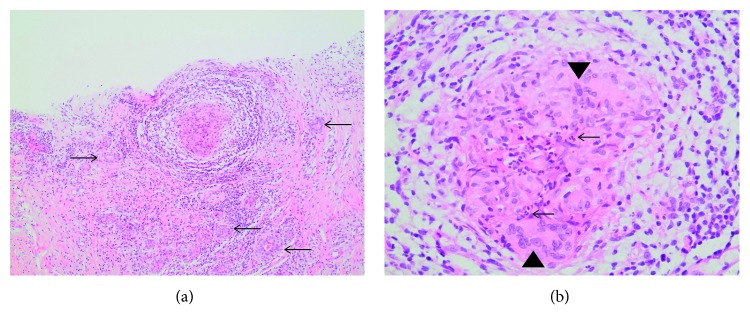
(a) Epithelioid granuloma and adjacent lobular acini surrounded by a lymphocytic inflammatory infiltrate (arrows) (H&E, 100x). (b) High-power view of the granuloma shows a mixture of epithelioid histiocytes (arrowheads) and lymphocytes and neutrophils (arrows) (H&E, 400x).

Core biopsy done in November of the same year showed multiple noncaseating epithelioid granulomas composed of epithelioid histiocytes, lymphocytes, neutrophils, and occasional multinucleated giant cells. Some granulomas contained neutrophils forming microabscesses with surrounding empty microcysts (Figure 1).

The Ziehl–Neelsen stain for acid-fast bacilli was negative. This leads to the diagnosis of idiopathic granulomatous mastitis which is a diagnosis of exclusion.

A repeat MRI done during June 2018 showed heterogeneous fibroglandular tissue with mild background enhancement. There are also numerous tiny rim-enhancing fluid collections in the left breast, the largest measuring 8 mm involving the upper inner and lower inner quadrants, some of which are fistulizing to the skin. Findings have regressed compared to the prior MRI.

As with the previous MRI, no enlarged axillary or internal mammary adenopathy was seen.

Those findings are consistent with biopsy-proven idiopathic granulomatous mastitis extensively involving the upper and lower inner quadrants of the left breast.

Our second patient is a 47-year-old woman, mother of 4 children with a past medical history of a left renal stone for which she underwent lithotripsy. Her past surgical history includes an appendectomy and cholecystectomy. She presented to the clinic in June 2016 as she felt a lump in her right breast along with some induration that has been present for the past 3 months. She was treated with augmentin, with no significant improvement.

On physical exam, there were 2 areas of induration associated with palpable masses that were tender to touch, but there were no palpable lymph nodes.

Ultrasound done in May 2016 showed a persistent large area of decreased echogenicity involving predominantly the upper outer quadrant of the right breast showing areas of fistulization to the skin and exhibiting increased vascularity. No suspicious lesions or enlarged lymph nodes were palpated on the left.

Core biopsy showed moderate acute and chronic inflammation predominantly around the ducts. Multiple, noncaseating granulomas were noted containing multinucleated giant cells. There was no evidence of malignancy. Methenamine stain (GMS) and acid-fast stains for fungi and mycobacteria were both negative.

A fine-needle aspirate (FNA) performed on an enlarged lymph node showed no metastatic carcinoma. Findings were consistent with a reactive lymph node.

She underwent a partial mastectomy in August 2016, and her wound was healing well. Moreover, the surgical pathology showed the same findings as the core biopsy which includes severe granulomatous mastitis with no evidence of malignancy. The patient would continuously follow up at the clinic, and her last appointment was in August 2017 where she presented with another nodule away from the scar site without any nipple discharge or erythema. The lesion is almost 1.3 cm big. It was shown that she has recurrent disease which is typical of granulomatous mastitis as it is chronic with high rates of recurrence.

## 3. Discussion

Idiopathic granulomatous mastitis (IGM) is a rare breast pseudotumor that most frequently occurs in parous women in their 3rd or 4th decade of life, who have a history of breastfeeding [9]. As seen in the cases discussed above, IGM has a very obscure presentation. It can present as an enlarging mass or as a hard irregular mass, which makes it very difficult to distinguish from breast cancer with just clinical and radiological findings. This will also lead to a delay in diagnosis. Our patients had to undergo several biopsies and imaging to reach a conclusive diagnosis [10]. Both of our patients had an ultrasound and MRI done, both of which were not sufficient to lead to a diagnosis. Breast cancer can be very aggressive, especially in this age group, and missing it can be unfortunate and fatal. That is why clinicians usually proceed with repeat biopsies. Therefore, the only way to reach a definitive diagnosis is by obtaining a biopsy [11]. Moreover, histology is a must as radiographic images are nonspecific [3].

It has been shown in the literature that the most common MRI finding in patients with IGM is nonmass enhancement, and the second most common one is enhancing masses [12, 13]. Aslan et al. reported that the most common patterns of nonmass enhancement in their case series were heterogeneous and clustered ring enhancement patterns [13]. Associated features on MRI, including skin thickening, skin retraction, and nipple retraction, have also been described on prior studies [12]. The ultrasound performed when the first patient presented also showed skin thickening, and there were skin retractions on physical exam.

As for mammography, the most common finding was focal symmetry, not associated with distortions or microcalcifications, whereas nodules with defined margins are less common [14]. Mammography was not done for both patients in the cases presented. The most common ultrasound findings include one or more irregular hypoechoic masses associated with increased echogenicity of the parenchyma [14]. The same finding was observed for our patient's ultrasound. Doppler study showed an increase in vascularization. Even though radiographic images give a lot of insights, these findings are nonspecific and can mimic the findings in the case of breast carcinoma [2].

As mentioned before, there yet to exist a noninvasive diagnostic tool to confirm the diagnosis of granulomatous mastitis and to help distinguish it from breast cancer. In one study, it has been shown that the IL-33 levels of patients with histological diagnosis of IGM have been found to be higher than those with breast cancer. Moreover, according to the ROC curves, IL-33 levels were both highly sensitive (93.75%) and specific (96%) in differentiating IGM from breast cancer. Therefore, IL-33 levels if used in conjugation with ultrasound and mammography can favor a diagnosis of IGM. However, the only method for definitive diagnosis at this point remains the use of biopsy [11].

There is not a lot of specific findings on radiographic images that directly lead to the diagnosis of GM [10]. In most of the studies, the first imaging modality used is ultrasound. Sonography is the most useful diagnostic method for the evaluation of IGM. This technique provides valuable insight for infectious conditions such as effusions, inflammation of parenchyma and fatty tissues, abscess formations, and fistula tracts. Such findings are key to differentiate the suspected lesions from malignancy [8]. Mammography has also been used in certain cases. FNA and core biopsy can also be used. It has been shown that core needle biopsy is diagnostic in 94.5% of patients while FNA is the only diagnostic in 39% of patients, which explains its debatable role [15]. Moreover, FNA may not always differentiate between GM and other granulomatous diseases of the breast. So, histology is the main foot of diagnosis either by core or open biopsy [16]. In the case of the first patient, an FNA was first performed followed by a core biopsy; however, in the case of the second patient, a core biopsy was performed without the use of FNA.

On histology, granulomatous mastitis is characterized by noncaseating granulomas in as well as around lobules and often in association with microabscess and fistula formation [1]. These findings were observed in our patients as well.

The noncaseating granulomas are made up of epithelioid histiocytes, neutrophils, and lymphocytes. Multinucleated giant cells were found in almost 78.5% of the case studies. Moreover, plasma cells were also encountered in almost all the specimens studied. Almost 53.9% of the cases showed cystic vacuoles rimmed with neutrophils in the center of the granulomas [17].

The difficulty to distinguish IGM from other similar diseases such as periductal mastitis and breast cancer can lead to management challenges [11]. There is a wide differential diagnosis that can range from an infective etiology including fungal or tuberculous infection to autoimmune, including sarcoidosis and granulomatosis with polyangiitis [1]. Even though granulomatous mastitis can have a wide differential, it is often regarded as idiopathic in most cases [1].

A possible association between corynebacteria infection and granulomatous mastitis was proposed, and there is growing evidence of an association between corynebacteria infection and a distinct pattern termed cystic neutrophilic granulomatous mastitis (CNGM), characterized by lipogranulomas consisting of clear spaces rimmed by neutrophils and surrounding granulomatous inflammation [4].

The distinct histological features of granulomatous inflammation with acute inflammation and cystic spaces should prompt careful search for rare Gram-positive bacilli within these spaces [9]. Microbiological investigation for *Corynebacteria*, including *C. kroppenstedtii*, should be instigated in the presence of these suggestive histological features.

Recognizing these histological clues and limitations of microbiological studies is imperative in providing an accurate diagnosis and in turn facilitating appropriate early antibiotic treatment for patients affected by this often debilitating disease process [9]. In one of the reported cases of recurrent granulomatous mastitis, it has been shown that *Corynebacteria kroppenstedtii* was the culprit. Not only did the patient have recurrent disease but also she developed an abscess in both breasts, the left followed by the right. *Corynebacteria* encompass a broad range of Gram-positive bacilli that are often components of the skin microbiome, which makes it difficult to distinguish the source of the bacteria as it might due to colonization, infection, or contamination [5]. In a 2002 retrospective study, it has been shown that 40% of isolates taken from histologically proven granulomatous lobar mastitis grew *C. kroppenstedtii* [5].

The mechanism of development of IGM is postulated to be initiated with ductal epithelial damage followed by transition of luminal secretions to the lobular connective tissue, local inflammation in connective tissue, macrophage and lymphocyte migration to the region, and local granulomatosis inflammatory response [18]. However, the initial trigger factor leading to the development of epithelial damage has not been determined. Trigger factors might include pregnancy, lactation, autoimmunity, hyperprolactinemia, and smoking, among others [18].

As mentioned before in the introduction, there is no universally accepted or practiced treatment for GM [19]. One of the most commonly used therapies includes surgical excision of the granuloma, drainage of the wound, and concomitant steroid therapy [19]. Other clinicians prefer the use of antibiotics, wide surgical resection, mastectomy, and immunosuppressants [20]. Other authors recommend wide excision combined with antibiotics if there is any evidence of infection. There is also a huge proportion of authors who conclude that steroid therapy is effective and resolution can be obtained without surgery [20].

Steroids as a primary treatment have proved to be beneficial [8]. Treatment with steroids is usually lengthy and can last for about 6 months; however, it is used for conservative management for its good success rates [10]. This treatment has been shown to help in shrinking the lesion both pre- and postoperatively in persisting masses [21]. Immunosuppressive drugs such as methotrexate and azathioprine have been considered as alternatives in case of recurrence or in case of deleterious side effects of prednisone [14].

There is a lot of heterogeneity when it comes to treatment patterns and preferences, and this can explain the high recurrence rates which can approach 50% [19]. Such a high rate of recurrence is alarming and proves that the current treatment modalities are suboptimal [20]. Moreover, the average time to recovery has been shown to be more than 1 year [1] with many patients undergoing many procedures and ending up with chronic and recurrent disease [1, 5].

The etiology of IGM remains unclear. It has been reported that various factors such as hormonal imbalance, as well as autoimmunity, can play a role in the disease process, maybe one that cannot be detected by the techniques currently available. Moreover, the only way to diagnose IGM is by a core biopsy as its radiographic findings are nonspecific. A lot of research still has to be done to determine the best treatment options associated with the lowest recurrence rates [22].
